# Yield Determination in Major Small Grain Crops in Response to Nitrogen Fertilization

**DOI:** 10.3390/plants14071017

**Published:** 2025-03-24

**Authors:** Milan Mirosavljević, Vojislava Momčilović, Vladimir Aćin, Bojan Jocković, Novo Pržulj, Goran Jaćimović

**Affiliations:** 1 Institute of Field and Vegetable Crops, 21000 Novi Sad, Serbia; vojislava.momcilovic@ifvcns.ac.rs (V.M.); vladimir.acin@ifvcns.ac.rs (V.A.); bojan.jockovic@ifvcns.ac.rs (B.J.); 2 Faculty of Agriculture, University of Banja Luka, 78000 Banja Luka, Bosnia and Herzegovina; novo.przulj@agro.unibl.org; 3 Faculty of Agriculture, University of Novi Sad, 21000 Novi Sad, Serbia; jgoran@polj.uns.ac.rs

**Keywords:** grain number, spike dry weight, fruiting efficiency, nitrogen response, small grain cereals

## Abstract

Small grain crops showed significant yield variation under different nitrogen fertilization treatments. Understanding factors influencing yield is crucial for optimizing productivity. This study assessed how nitrogen fertilization affects grain number, grain weight, and other yield components in triticale, wheat, six-rowed, and two-rowed barley across multiple locations and seasons. Key traits, including grain number per spike, spike number per unit area, and spike dry weight, were analyzed. Triticale cultivars achieved the highest average grain yield (GY) of 8709 kg ha−1, significantly outperforming wheat (7656 kg ha−1) and six-rowed barley (7676 kg ha−1), particularly under high nitrogen (100 kg N) fertilization, where triticale reached 9184 kg ha−1. Grain number per unit area (GN) was strongly positively correlated with GY across all crops, with the highest GN values observed in triticale (21,937) under 100 kg N fertilization. Fruiting efficiency (FE) and spike dry weight at anthesis (SDWa) showed crop-specific relationships with GN, with the strongest association between GN and SDWa observed in triticale, while FE exhibited the highest values in wheat (112.3) and the lowest in two-rowed barley (82). Triticale’s superior yield was linked to greater grain number and spike dry weight, while wheat’s high fruiting efficiency contributed to its performance. Despite its high spike number and spike dry weight, two-rowed barley had lower grain number per spike, limiting its yield. Nitrogen fertilization improved yield components across all crops, though trade-offs between grain weight and other traits were observed. These findings provide insights for breeders and agronomists in optimizing small grain production.

## 1. Introduction

Small grain cereals play an important role as major winter crops in the Pannonian basin and neighboring European regions, providing valuable alternative to major spring crops such as maize, sunflower, and soybeans [1]. As global demand for cereal grains is projected to increase in the coming decades, there is consensus that accelerating yield potential increases is imperative to achieving sustainable production improvement. However, the current rates of genetic gains in wheat, below 1% per year in many countries, fail to meet the projected cereal demand [2]. This coincides with a reduction in global productivity per capita due to increasing population growth. Therefore, a deeper understanding of crop yield-related physiological traits could aid in achieving the necessary rates of genetic gains.

Major winter small grain crops, including wheat, barley, and triticale, share similar agronomic niches, characterized by comparable soil cultivation techniques, sowing dates, fertilization, and other significant agricultural management practices. However, slight differences in yield determination and resource use efficiency exist among these small grain crops. For instance, the increased grain yield (GY) of triticale has been associated with higher radiation use efficiency, increased biomass at anthesis and maturity, and a greater grain per spike number compared to bread wheat [3]. Moreover, winter barley, and more recently, triticale, have been characterized as crops that efficiently utilize resources and are often cultivated on less fertile soils or with the limited application of agronomic practices, such as nitrogen fertilization, while winter wheat has been favored for production under high-yield conditions [4]. Recognizing these slight differences is crucial for adjusting cultivation methods and maximizing crop productivity.

Nitrogen (N) fertilizer plays a crucial role in the fertilization of small grain crops and significantly influences GY determination. Small grains such as wheat, barley, and triticale have high N requirements during their growth stages, particularly during tillering and stem elongation [5]. Adequate N availability promotes vegetative growth, enhances photosynthesis, and increases the number and size of grains per spike. Moreover, N fertilization positively impacts grain quality parameters such as protein content and grain weight. However, excessive N application can lead to lodging, disease susceptibility, and environmental pollution through nitrogen leaching and emissions [6,7]. Therefore, proper N management practices, including precise timing, rates and application methods, are crucial for optimizing GY while minimizing negative environmental impact.

In small grains, GY results from two primary factors: the number of grains per unit area (GN) and the average grain weight (GW). Therefore, it can be concluded that improvements in either of these two factors should lead to higher GY. The existence of a trade-off between average GW and GN represents a challenge to GY increase. This trade-off may be associated with various factors, such as source limitations during grain filling [8], or the positioning of smaller grains at the distal ends of spikes or spikelets, which have lower weight potential [9]. This trade-off may lead to a decline in overall GY if the decrease in GW outweighs the increase in GN [10].

Moreover, the relative importance of GW and GN in determining yield is not equal. The GY of various cereal crops is significantly more influenced by the GN and is largely unrelated to GW. The close relationship between GY and GN is attributed to the extensive plasticity of GN, whereas average GW tends to be a relatively stable trait [11]. Numerous studies on historical GY gain in various small grain crops have indicated that GN was the primary driver of breeding progress in GY. On the other hand, changes in GW were less pronounced, often showing stagnation during past breeding periods [9]. Therefore, to achieve further increases in GY, it is crucial to maintain focus on the rise in GN per unit area. Additionally, GN could be further dissected into its numerical components, such as spike number per unit area (SN) and grain number per spike (GNS). However, given that GN is a highly complex trait influenced by genotype, environment, management practices, and their interactions, achieving precise control in determining GN requires further investigation. Various fertilization levels and application methods are suggested to meet the demands of different yield components and reduce N losses [12]. Additionally, N application could further disrupt the relationship between GW and GN, as it has a positive impact on GN per square meter while reducing the average GW [13]. This complication makes it challenging to precisely determine target N applications.

In this study, we analyzed the GY determination of different winter cereal under varying N fertilizer levels across different locations and growing seasons. Assuming that different cereal crops have specific yield determination mechanisms, our objective was to identify the mechanisms related to GY determination in wheat, barley, and triticale, analyzing the physiological strategies and possible trade-offs.

## 2. Results

### 2.1. Weather Conditions

The three growing seasons (2019/20, 2020/21, and 2021/22) had distinct climatic patterns across the locations. 2019/20 was marked by moderate precipitation but notably low rainfall in April (11 mm in Sombor and 14 mm in Novi Sad), a critical period for grain number (GN) formation, which likely contributed to the reduced average yields observed that year. In contrast, 2020/21 had more evenly distributed rainfall, particularly during the October–May period, supporting higher GN and overall yield stability. However, 2021/22 experienced extreme drought in March (1 mm in Novi Sad) but higher rainfall in June, which may have benefited late-season grain filling. Temperature patterns also varied, with 2019/20 having milder spring temperatures, while 2020/21 and 2021/22 were characterized by colder winters and warmer springs. The most balanced climate in 2020/21, with consistent rainfall and moderate temperatures, correlated with higher GN per spike and more stable yields compared to the other two years.

### 2.2. Grain Yield, Weight, and Number

**Figure 1:**
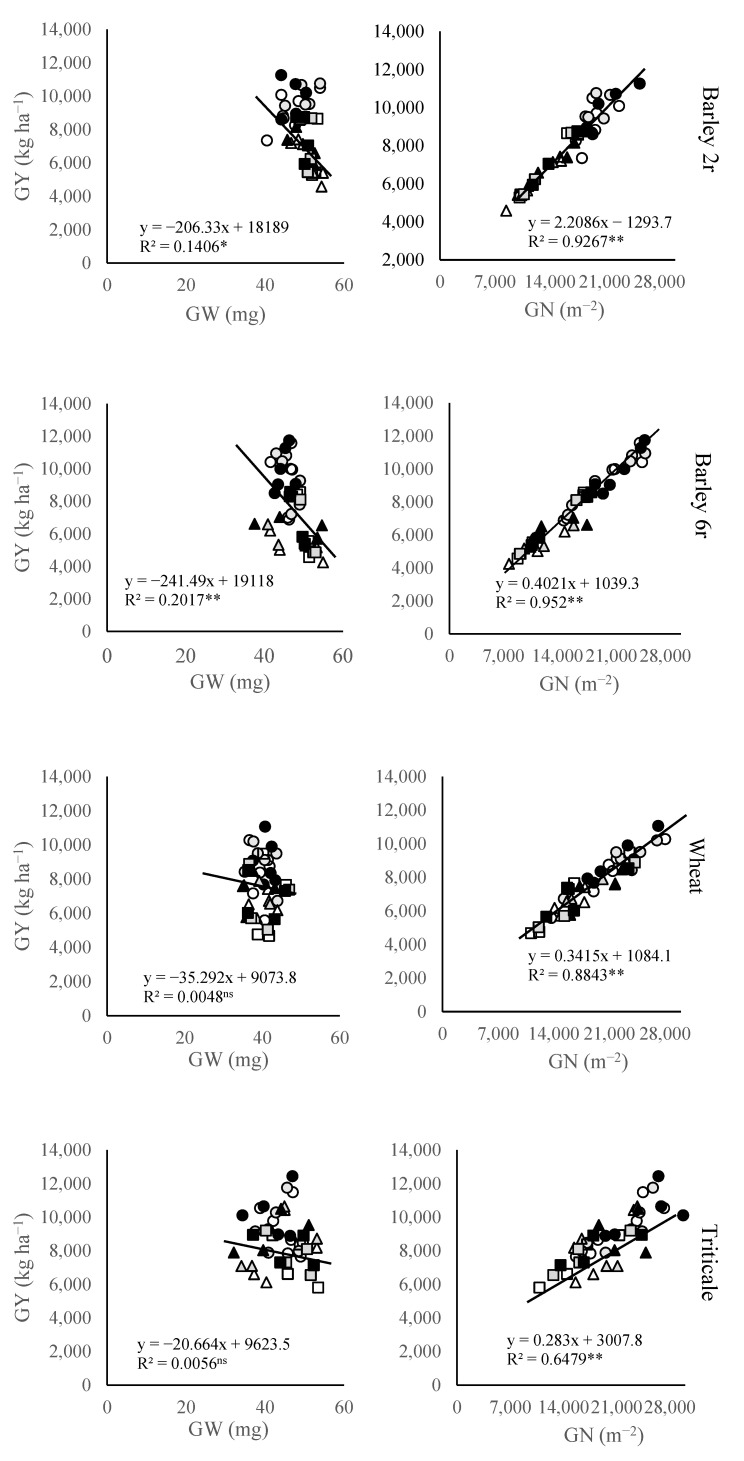
Relationship between small grains grain yield (GY) with grain weight (GW) and grain number per unit area (GN) at three locations during two growing seasons. Symbols for each location are □ Sombor, ○ Novi Sad, and ∆ Sremska Mitrovica, where open symbols represent control 0 N treatment, gray symbols represent 50 N treatment, and dark symbols represent 100 N treatment. *—Significant at *p* < 0.05; **—Significant at *p* < 0.01; ns—Not significant.

Results from our trials indicate that the grain yield (GY) of small grain crops varied across N fertilization treatments and combinations of locations and growing seasons (Figure 1; Table S1). On average, triticale cultivars (8709 kg ha^−1^) tended to have higher GY compared to other small grains, while winter wheat (7656 kg ha^−1^) and six-rowed barley (7676 kg ha^−1^) demonstrated the lowest GY. Under limited N conditions, two-rowed barley, six-rowed barley, wheat, and triticale achieved 7661, 7396, 7256, and 8357 kg ha^−1^, respectively. With the application of 50 kg N, the average GY for two-rowed barley, six-rowed barley, wheat, and triticale increased to 7974, 7520, 7802, and 8586 kg ha^−1^, respectively. The highest average GY was recorded under 100 N conditions, with values of 8357, 8110, 7910, and 9184 kg ha^−1^ for two-rowed barley, six-rowed barley, wheat, and triticale, respectively.

The grain weight (GW) had notable variation among different crops, locations, and N fertilization levels. Among the crops, wheat displayed the lowest GW values (40.2 mg), while the highest average GW values were observed in two-rowed barley (49.4 mg). Under rising fertilization levels, in wheat and triticale, GW showed an opposite response to N supply compared to GN, as high N supply led to a slight reduction in GW. However, compared to the unfertilized control in both barley crops, GW slightly increased under medium fertilizer levels but decreased under the 100 N treatment. Furthermore, a significant negative relationship was observed between GW and GY in both two-rowed and six-rowed barley crops, while this relationship was absent in wheat and triticale.

The grain number per unit area (GN) showed notable variation among different crops, locations, and N fertilization levels. Among crops, the lowest GN values were observed in two-rowed winter barley (16,369) and six-rowed winter barley (16,502), while the highest average GN values were recorded in triticale (20,150), followed by wheat (19,247). Notably, the highest GN for each of the small grain crops (17,325 in two-rowed winter barley, 17,695 in six-rowed barley, 19,891 in wheat, and 21,937 in triticale) was achieved with 100 N fertilization treatments. This represented a notable increase compared to unfertilized treatments, where GN values were notably lower (15,791 in two-rowed barley, 15,817 in six-rowed barley, 18,097 in wheat, and 18,806 in triticale). Overall, the variation in GY of triticale, wheat, two-rowed barley, and six-rowed barley was positively correlated with GN.

### 2.3. Grain Number Subcomponents

**Figure 2:**
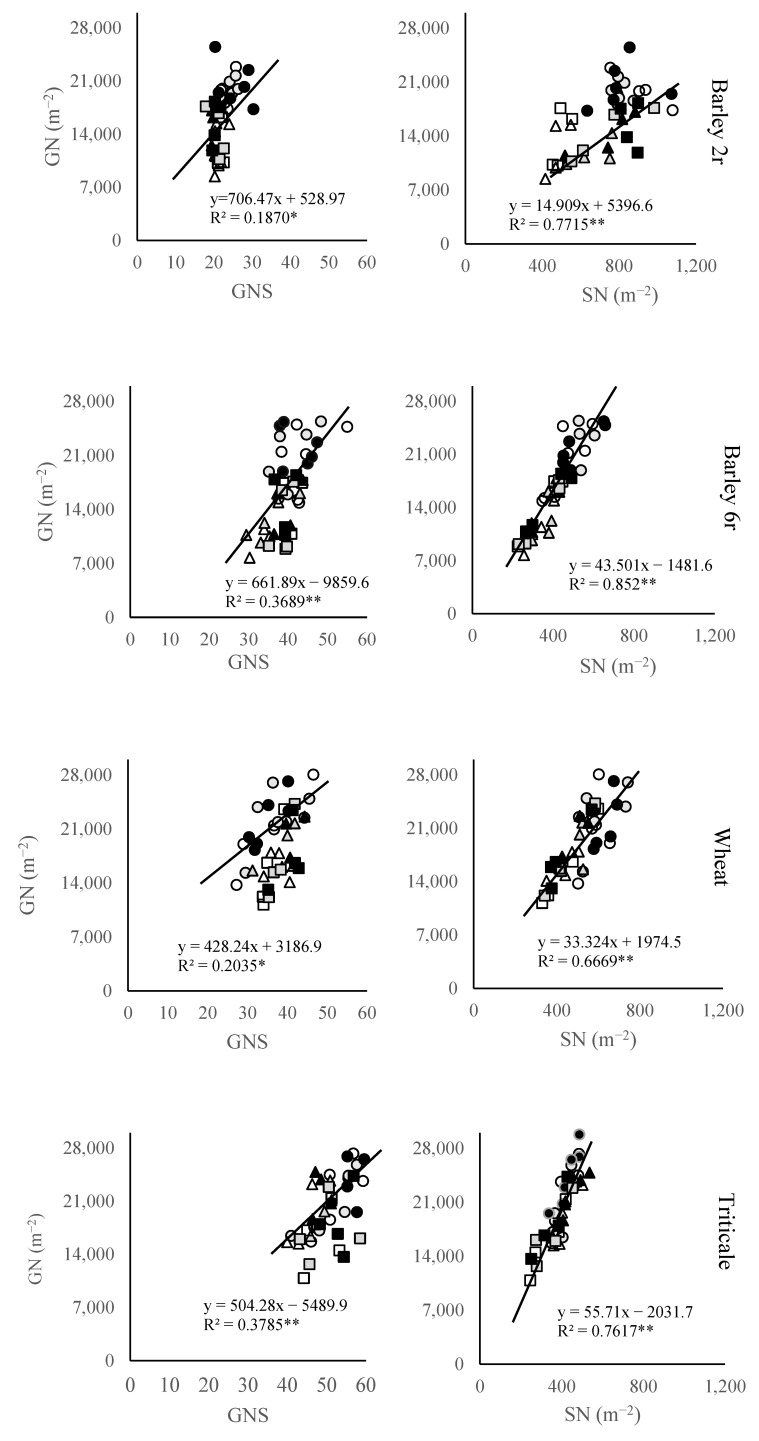
Relationship between small grains spike number per unit area (GN) with grain number per spike (GNS) and spike number per unit area (SN) at three locations during two growing seasons. Symbols for each location are □ Sombor, ○ Novi Sad, and ∆ Sremska Mitrovica, where open symbols represent control 0 N treatment, gray symbols represent 50 N treatment, and dark symbols represent 100 N treatment. *—Significant at *p* < 0.05; **—Significant at *p* < 0.01.

The grain number per spike (GNS) varied across different crops, locations, and N fertilization levels (Figure 2). Among these crops, two-rowed winter barley had the lowest GNS values (22.4), while triticale showed the highest GNS (50.8). With the application of N fertilization, GNS tended to increase in wheat, and triticale. In two-row barley, there was an increase in GNS with an increase in fertilization from 0 N to 50 N, while a further increase to 100 N did not result in a rise in GNS but rather led to a slight decrease. However, in six-rowed barley, there was no clear trend in GNS changes, with similar values observed under both control and high N application. Additionally, a significant positive association was noted between GNS and GN in all studied crops, with the strongest relationship reported in triticale and six-rowed barley.

Conversely, GN showed a higher correlation with spike number per unit area (SN) variation in the studied crops. The most pronounced relationship between these two traits was observed in six-rowed (R^2^ = 0.85) and two-rowed barley cultivars (R^2^ = 0.77). Among these crops, two-rowed winter barley cultivars displayed the highest SN at 736, while six-rowed barley (413) and triticale (398) exhibited notably lower values for this trait. In most instances, N application resulted in a significant increase in SN across the studied crops compared to the control, except for the 50 N application in six-rowed winter barley. The highest SN values were recorded under the highest N treatment, reaching 764, 437, 528, and 417 in two-rowed barley, six-rowed barley, wheat, and triticale, respectively.

**Figure 3:**
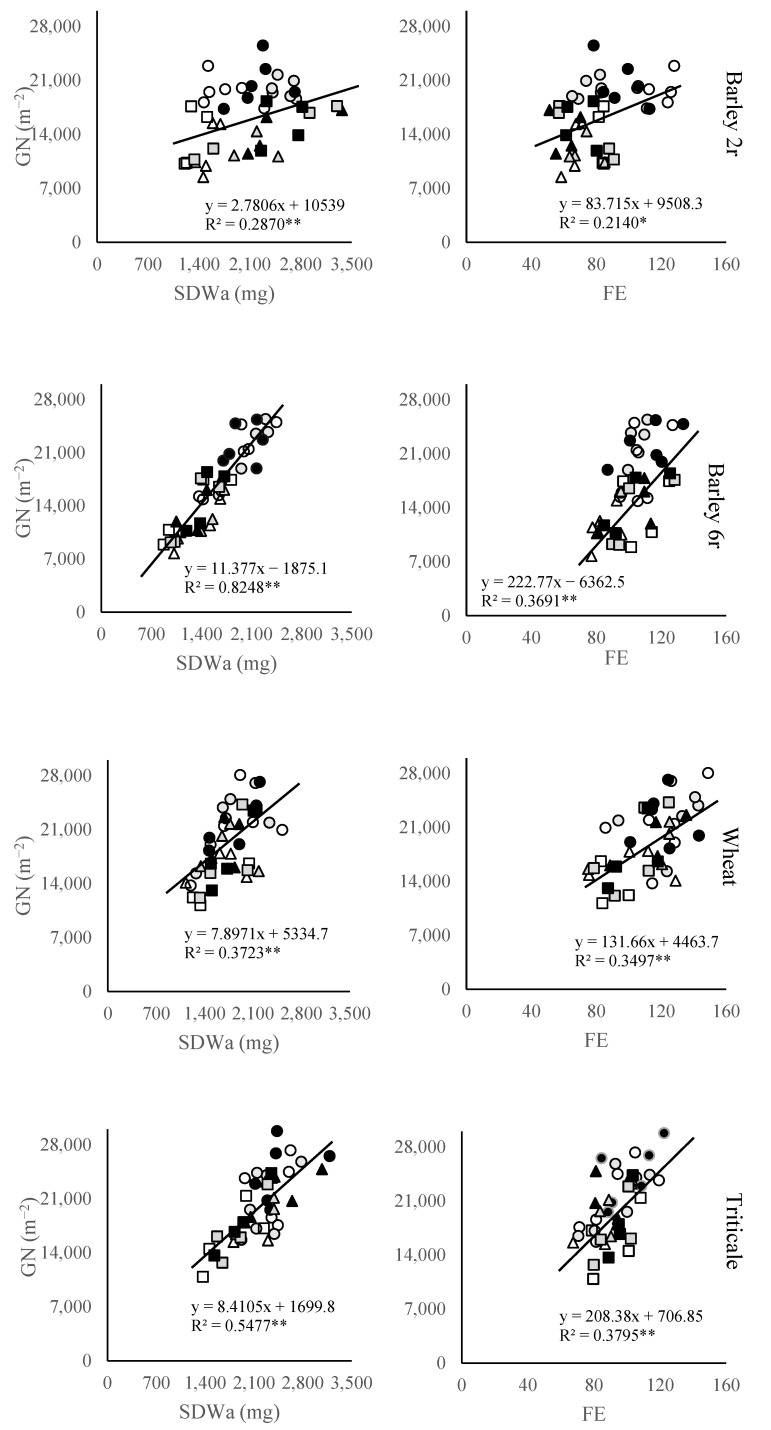
Relationship between small grains spike number per unit area (GN) with spike dry weight at anthesis (SDWa) and fruiting efficiency (FE) at three locations during two growing seasons. Symbols for each location are □ Sombor, ○ Novi Sad, and ∆ Sremska Mitrovica, where open symbols represent control 0 N treatment, gray symbols represent 50 N treatment, and dark symbols represent 100 N treatment. *—Significant at *p* < 0.05; **—Significant at *p* < 0.01.

Next to the GNS and SN, other GN subcomponents have been analyzed (Figure 3). Of the crops, triticale (2194 mg) and two-rowed winter barley (2097 mg) cultivars displayed a higher spike dry weight at anthesis (SDWa) compared to wheat (1762 mg) and six-rowed barley (1615 mg). SDWa response to the N fertilization followed a similar trend as a SN, showing an almost linear increase with increasing N treatments in six-rowed barley, wheat, and triticale. Moreover, the relationship between SDWa and GN varied among studied crops, showing the highest relationship in six-rowed barley and lowest in two-rowed barley.

In general, grain number (GN) exhibited a weaker correlation with fruiting efficiency (FE) compared to spike dry weight at anthesis (SDWa). The closest association between GN and SDWa was observed in triticale and six rowed barley, while in two-rowed barley, the relationship between FE and GN resembled that of SDWa and GN. Wheat cultivars tended to display higher FE values (112.3) compared to other crops, particularly in contrast to two-rowed winter barley, which had the lowest FE (82). Across all crops, there was a notable increase in FE as a result of N treatments compared to unfertilized treatments.

**Figure 4:**
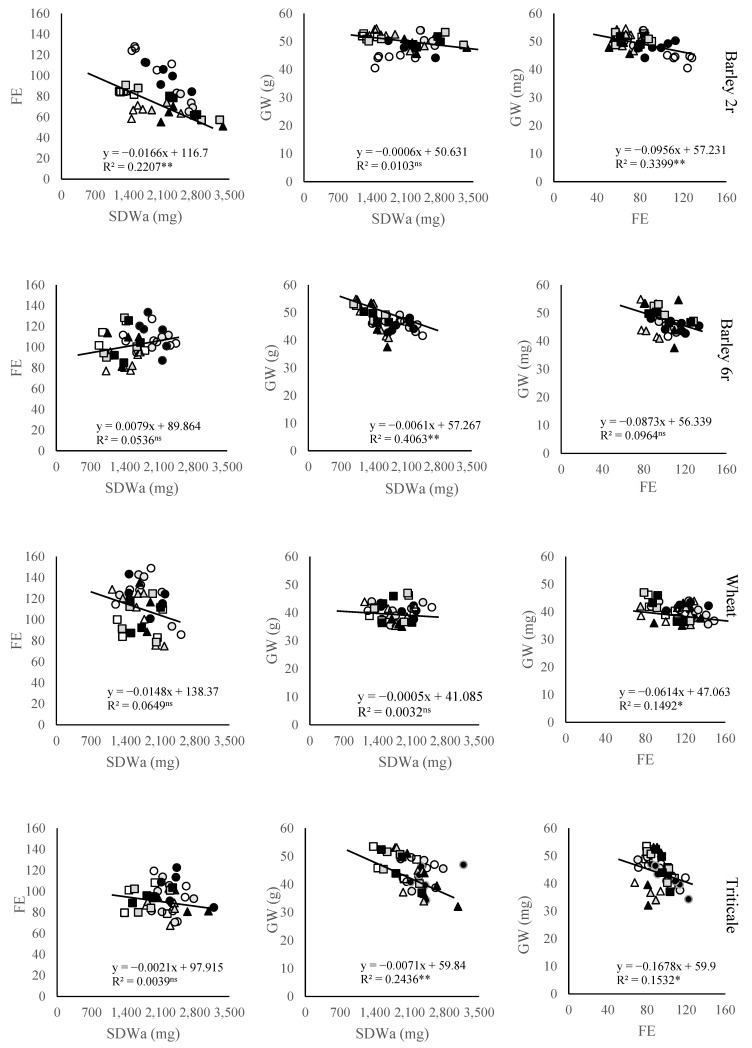
Relationships between small grains spike dry weight at anthesis (SDWa), fruiting efficiency (FE), and grain weight (GW) at three locations during two growing seasons. Symbols for each location are □ Sombor, ○ Novi Sad, and ∆ Sremska Mitrovica, where open symbols represent control 0 N treatment, gray symbols represent 50 N treatment, and dark symbols represent 100 N treatment. *—Significant at *p* < 0.05; **—Significant at *p* < 0.01; ns—Not significant.

The relationships between fruiting efficiency (FE) and spike dry weight at anthesis (SDWa), as well as between FE and grain weight (GW), varied significantly among the studied small grain cereal crops (Figure 4). In six-rowed barley and triticale, GW showed a statistically significant negative relationship with SDWa, while in two-rowed barley, wheat, and triticale, it was significantly negatively related to FE. The association between SDWa and FE was generally absent in most crops, except in two-rowed barley, where a negative relationship was observed.

## 3. Discussion

### 3.1. Grain Yield Variation

The results of this study indicate notable variations in GY among studied small grain crops, with N fertilization playing a significant role in determining GY. On average, triticale demonstrated the highest GY (8709 kg ha^−1^) across all N treatments, while wheat (7656 kg ha^−1^) and six-rowed barley (7676 kg ha^−1^) consistently showed lower GY. The GY difference between six-rowed barley and wheat was less pronounced, which aligns with previous research investigating the performance of wheat and barley across various environments, particularly in Mediterranean climates [14,15]. The advantage of barley over wheat is often attributed to earlier heading and flowering [16], which can mitigate the adverse effects of high temperatures and drought occurring during reproductive phases. However, in the Pannonian Plain, the anthesis dates of wheat and six-rowed barley are nearly identical, while two-rowed barley flowers a few days earlier than wheat, six-rowed barley, and triticale [11,17]. Consequently, the slightly higher yields of two-rowed barley compared to six-rowed barley and wheat may be partly attributed to these phenological differences.

Moreover, our results suggest that triticale has a higher GY potential under the conditions of the Pannonian Plain, and its role in crop rotation within these conditions should receive more attention. Previous studies have also highlighted the superiority of triticale compared to other small grains such as wheat [18], durum wheat [19], and barley [20]. This advantage is primarily attributed to the unique plant architecture of triticale, which results in increased biomass at anthesis and maturity, coupled with higher radiation use efficiency, likely due to improved light distribution within the canopy [3]. The positive response of GY to increased N application was observed across all crops, with the highest GY recorded under 100 kg N ha^−1^ (8357 kg ha^−1^ for two-rowed barley, 8110 kg ha^−1^ for six-rowed barley, 7910 kg ha^−1^ for wheat, and 9184 kg ha^−1^ for triticale). This finding aligns with prior studies indicating that N availability is a key limiting factor in crop productivity [21,22]. However, the level of GY increase varied among crops, with triticale showing the most pronounced response, followed by two-rowed barley. In contrast, six-rowed barley and wheat exhibited more modest increases under high N conditions, potentially reflecting differences in N uptake efficiency or the ability to convert absorbed N into GY. The importance of crop-specific responses to N fertilization and climatic conditions has been reported in previous research. Triticale’s superior yield under high N conditions is linked to its efficient nitrogen use efficiency (NUE) and greater biomass allocation to reproductive structures [23]. Triticale is characterized by early root growth and increased transpiration efficiency, which contribute to biomass accumulation. Its vigorous early root development helps the crop capture more nitrogen that would otherwise be leached beyond the root zone [24,25].

### 3.2. Grain Number and Its Components

In small grain crops, GY is determined by both grain number (GN) and grain weight (GW). Numerous studies emphasize the major role of GN in determining GY [26,27]. According to the results of this study, wheat and triticale (20,150) showed higher GN per unit area compared to barley (two-rowed winter barley—16,369; six-rowed winter barley—16,490), while barley had a slightly higher GW. These differences are primarily explained by variations in spike architecture and the number of florets. Generally, wheat and triticale produce more fertile florets than two-rowed barley, resulting in greater plasticity in GN per spike for wheat and triticale [28]. On the other hand, the greater GW observed in barley compared to wheat and triticale is primarily due to morphological differences among these species. Additionally, breeding programs in barley have prioritized the development of cultivars with a higher proportion of grains suitable for the malting industry [29]. Furthermore, advances in genetic research have shed light on the mechanisms regulating GNS and SN. Recent studies have identified key genes and pathways involved in spikelet initiation and fertility in cereals, which may explain differences in grain number per spike (GNS) between species. These genes regulate diverse processes including transcription, hormone metabolism, and post-transcriptional modifications, influencing inflorescence architecture and spikelet development [30]. Understanding the genetic factors controlling floral development and inflorescence architecture to enhance grain yield in crops like wheat and barley [31]. The significant increase in GY and GN with rising N application highlights the importance of N in enhancing the productivity of small grain crops. Although both GN and GY improved with higher N levels, this increase often coincided with a reduction in GW, particularly in two- and six-rowed barley under 100 N. This trade-off is well-documented in cereal crops, where GN rise is primarily driven by an increase in secondary spikes and grains located in the distal and upper parts of the spike, which generally have limited potential for higher grain weight [28,32].

In terms of grain number subcomponents, grain number per spike (GNS) and spike number per unit area (SN) varied significantly among the studied crops. Triticale had the highest average GNS (51.4), contributing to its higher GY performance, while two-rowed barley showed the lowest GNS (22.4). The observed increase in SN under N fertilization, particularly in two-rowed barley, highlights the critical role of SN in improving GN and yield. Conversely, the more modest increase in SN observed in wheat and triticale suggests that these crops depend on a simultaneous increase in both GNS and SN to enhance GY under higher N conditions. GN per unit area was positively associated with both GNS and SN, although the association was stronger with SN, which is consistent with previous studies [33,34]. The slightly stronger association between GN and SN in both barley types compared to wheat can be attributed to the superior tillering capacity of barley, especially in two-rowed barley [17]. However, the high tillering capacity and greater SN of two-rowed barley could not compensate for its lower GNS compared to wheat and triticale. On the other hand, triticale and six-rowed barley showed a balanced strategy to determine GN, leveraging both GNS and SN. The weaker association between GN and GNS in wheat and two-rowed barley can be explained by a trade-off between GNS and SN [35]. This trade-off reflects a fine-tuning mechanism, where SN acts as a coarse regulator of GN, while GNS serves as a fine-tuning regulator, driven by genotypic differences.

The further analysis of GN subcomponents, including fruiting efficiency (FE) and spike dry weight at anthesis (SDWa), revealed additional insights into the GY formation strategies among the studied small grain cereals. Among these crops, triticale (2191 mg) and two-rowed winter barley (2097 mg) demonstrated higher average SDWa compared to wheat (1780 mg) and six-rowed barley (1616 mg), indicating a stronger potential for biomass allocation to reproductive structures and aligning with their high SN values [36]. Notably, the response of SDWa to N fertilization followed a similar pattern as SN, with a nearly linear increase observed in six-rowed barley, wheat, and triticale, suggesting that both SDWa and SN are critical components in determining GN under higher N availability. Also, FE increased due to N fertilizer application, indicating that N availability can enhance floret development and survival, thereby improving FE [37]. Optimal N application enhances assimilate partitioning to developing spikes, improving both FE and GN [38]. Higher FE can be achieved through the increased allocation of assimilates to developing florets or reduced floret demand for normal development. However, potential trade-offs between FE and grain weight must be considered when breeding for this trait [39]. The presence of genetic variability in FE among elite germplasm, along with recent advances in understanding its physiological and genetic basis [40], makes it a valuable selection criterion for wheat breeding programs. The relationship between SDWa and GN varied significantly among the studied crops. Six-rowed barley exhibited the strongest association, reflecting its reliance on SDWa for GN determination. In contrast, two-rowed barley displayed the weakest correlation, indicating that its GN formation is less dependent on SDWa. Despite its high SN and SDWa, two-rowed barley had the lowest FE, which was insufficient for compensating for its lower GNS. The negative relationship between FE and SDWa in two-rowed barley suggests that gains in FE were less relevant to increasing GY in this crop, although higher SDWa can accumulate more biomass, they may not always convert this biomass into grains efficiently. On the other hand, wheat cultivars displayed the highest FE values, likely due to their efficient allocation of assimilates to grain production. FE is positively associated with grain number per spike and is primarily determined by grain number per fertile spikelet [41]. Genome-wide association studies have identified several marker–trait associations for FE and related traits, offering potential for marker-assisted selection [41]. FE is also influenced by nitrogen (N) availability, with efficient N use and carbon partitioning during grain filling contributing to higher FE [42,43]. The relationships between FE and SDWa, as well as between FE and GW, further highlight the complexity of GY determination. In six-rowed barley and triticale, a significant negative correlation between GW and SDWa was observed, suggesting that increases in SDW may lead to more grains per spike diluting individual grain weights. Similarly, a significant negative correlation between GW and FE was noted in two-rowed barley, wheat, and triticale. This indicates that higher efficiency in transforming pre-anthesis spike weight into grain number per spike limited the availability of resources for producing individual grains with higher weight [44]. Interestingly, while most crops exhibited no significant relationship between SDWa and FE, a negative association was observed in two-rowed barley, highlighting its unique yield formation dynamics. The lack of a strong association between FE and SDWa in wheat, triticale, and six-rowed barley suggests that changes in one component will not be offset by trade-offs in the other. This aligns with studies proposing that FE and SDWa are relatively independent traits [45,46]. There are also differences in SDWa and FE control. While FE in wheat is primarily under genetic control, spike dry weight is significantly influenced by environmental factors [40]. However, the negative correlation observed in two-rowed barley between these two components could be attributed to its low plasticity in GNS and high tillering capacity. An increase in SN and SDWa in this crop may lead to a further reduction in GNS and fruiting efficiency, reflecting the challenges in balancing yield components in two-rowed barley. These findings deepen our understanding of the physiological constraints influencing yield formation in small grain cereals and underscore the need for crop-specific management strategies.

## 4. Materials and Methods

### 4.1. Experimental Site and Design

**Figure 5:**
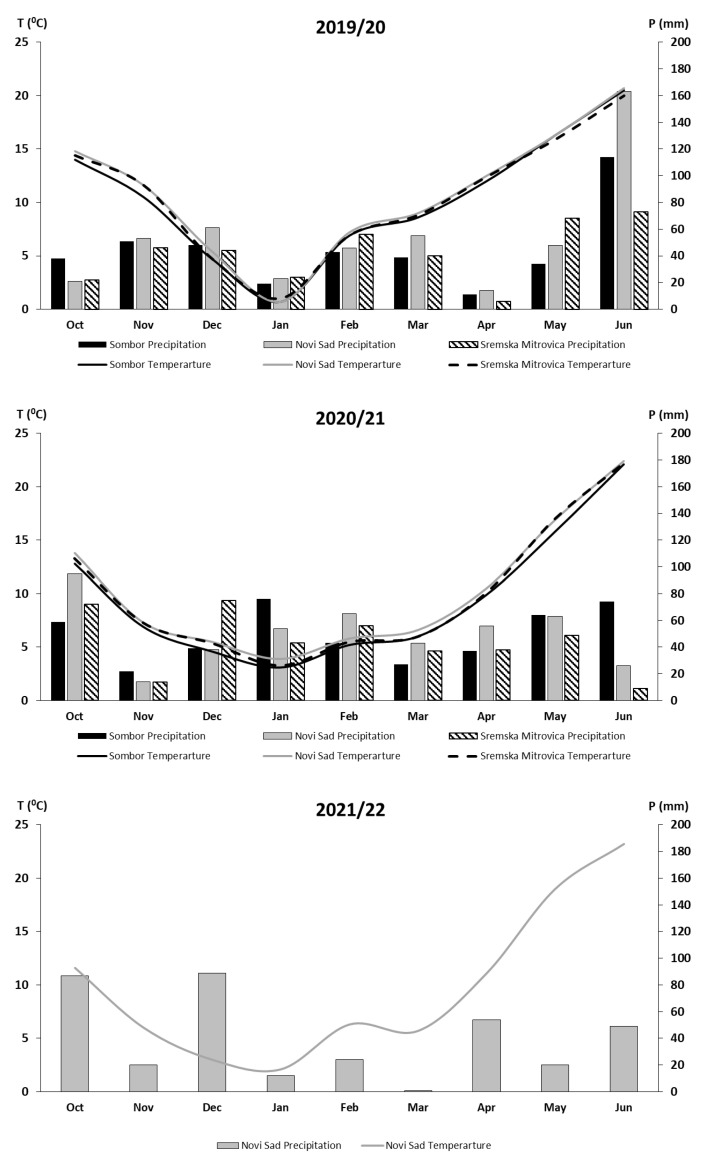
Average monthly temperatures and precipitation for each growing season (2019/20, 2020/21 and 2021/22) at three locations: Novi Sad, Sombor, and Sremska Mitrovica.

The experiment was conducted over two growing seasons, 2019/20, 2020/21, and 2021/22, at three locations in northern Serbia within the Vojvodina region (southern Pannonian Plain). The trials were established at the experimental field of the Institute of Field and Vegetable Crops in Novi Sad (NS) in 2019/20, 2020/21, and 2021/22 and the research fields of the Agricultural Extension Service in Sremska Mitrovica (SM) and Sombor (SO) in 2019/20 and 2020/21. Details of the environmental conditions and characteristics of the experimental sites are presented in Table 1 and Figure 5. This study aimed to compare the performance of various small-grain cereals across a wide range of environmental conditions, through the combination of different growing seasons, locations, and N fertilization levels.

Treatments within each location consisted of a factorial combination of (a) winter wheat, triticale, two-rowed barley, and six-rowed barley and (b) three levels of N fertilization: low (0 kg N ha^−1^), moderate (50 kg N ha^−1^), and high (100 kg N ha^−1^). The treatments were arranged in a split-plot design with four replications, where the main plot is the N fertilization level and the subplot represents the cultivar. To minimize potential N losses, the fertilizer was applied in two stages: after crop emergence (GS11-12) [47] (25 kg N ha^−1^ for the moderate level and 50 kg N ha^−1^ for the high level) [19] and prior to the start of the stem elongation period (GS30) (25 kg N ha^−1^ for the moderate level and 50 kg N ha^−1^ for the high level), using ammonium nitrate (34%). All N applications were performed on the same day for all cultivars.

This study evaluated eight cultivars of small-grain cereals, including two winter wheat cultivars (*Triticum aestivum* L.), two triticale cultivars (× *Triticosecale* spp. Wittmack), two two-rowed barley cultivars (*Hordeum vulgare* subsp. *distichum* L.), and two six-rowed barley cultivars (*Hordeum vulgare* subsp. *hexastichon* L.). Although two-rowed and six-rowed barley belong to the same species, they were analyzed separately due to notable differences in grain yield determination. Despite significant genotypic variation within each species, two representative cultivars were selected per species to enable the efficient management of the trials across diverse locations and N fertilization levels. The chosen cultivars reflect widely grown and well-adapted varieties developed for the Pannonian Plain (Table S2). The winter wheat cultivars Simonida and NS 40S are widely cultivated in Serbia and the surrounding region, serving as standards in national registration trials. For two-rowed barley, Novosadski 525 and Novosadski 565 are prominent local varieties, while six-rowed barley cultivars Nonius and Rudnik are widely grown in Serbia, with Rudnik also serving as a national standard. The triticale cultivar Odisej is recognized as a standard in national registration trials, while NS Paun represents a newer high-yielding cultivar in production.

The trials were carried out during the recommended planting periods in October 2019 and 2020, with a target density of 450 viable seeds per square meter, a standard seeding rate for the agroecological conditions of the southern Pannonian Plain. Each experimental plot measured 5 m in length and 1 m in width, consisting of 10 rows. Soil samples were collected from the 0–60 cm depth before sowing to assess soil mineral nitrogen [48], phosphorus (P), and potassium (K) (Egner–Riehm) levels, with results detailed in Table 1. The soil at the experimental sites was classified as chernozem (according to the IUSS Working Group WRB, 2014). Balanced P and K fertilization was applied before sowing to prevent deficiencies. The preceding crops at the experimental sites were legumes. After the application of P and K fertilizers, the plots were plowed and prepared for sowing using standard pre-sowing tillage practices. Weeds (750 g kg^−1^ tribenuron-methyl + 333 g L^−1^ fluroxypyr) and diseases (80 g L^−1^ Cyproconazole + 200 g L^−1^ Picoxystrobin) were managed as needed in spring using recommended chemical treatments.

### 4.2. Crop Measurements and Data Analysis

Crop growth stages were regularly monitored during the growing seasons using the decimal code of Zadoks. Anthesis (GS65) and physiological maturity (GS89, defined as 50% yellowing of the peduncle) were recorded for each cultivar, and sampling schedules were adjusted to align with cultivar- and species-specific phenology. Biomass sampling was conducted at both anthesis and maturity by harvesting two 1 m long samples from the central rows of each plot to avoid border effects [9,17]. The harvested plant material was oven-dried at 60 °C for 48 h before being weighed. At anthesis, spikes were separated from the remaining canopy material. The number of spikes per square meter was determined, and the spike dry weight at anthesis (SDWa) was calculated and expressed as spike dry weight (mg) per m^2^. At maturity, plant samples were separated into spikes with grains and the remaining canopy. From these samples, the number of spikes per square meter (SN) and the number of grains per spike (GNS) and grain number per unit area (GN) were determined. Grain weight (GW) was calculated from a randomly selected subsample of 200 grains. Plots were mechanically harvested, and grain yield (GY) was calculated on a per-plot basis. The grain weight from the 1 m samples was added to the grain weight obtained from the combine-harvested plot. The final grain yield (t ha^−1^) was adjusted to 13% moisture content. Fruiting efficiency (FE) was calculated as the ratio of the number of grains per spike to the spike dry weight at anthesis.

Prior to analysis of variance (ANOVA), the Shapiro–Wilk test was used to assess the normality of the data distribution, while Bartlett’s test was applied to evaluate the homogeneity of variances. ANOVA for the three factors (location × year, nitrogen, and crops) was performed, and for each treatment, the mean difference was analyzed using the Tukey test at a 0.05 probability level. Regression analysis was used to identify the relationship between the species and the nitrogen used by them. Statistical analyses, including ANOVA, the Shapiro–Wilk test for normality, Bartlett’s test for homogeneity of variances, and regression analysis, were conducted using the R computing language (version 4.4.1).

## 5. Conclusions

This study revealed significant variation in GY and its components across small grain cereals, highlighting the superior performance of triticale under both nitrogen treatments. Triticale’s GY advantage was attributed to its higher GN and SDWa, demonstrating its superior biomass allocation and reproductive efficiency. The superior performance of triticale under high N conditions suggests its potential as a key crop in rotations within the Pannonian Plain, particularly in systems aiming for high biomass and grain yield. Two-rowed barley, despite its higher SN and SDWa, showed lower FE and GNS, limiting its GY potential. Interestingly, wheat exhibited the highest FE values, underlining its efficient assimilation to grain production and suitability for environments where efficient resource allocation is critical. The N fertilization consistently improved GY and reproductive traits across all crops, with notable increases in GN, SDWa, and FE, particularly in triticale and six-rowed barley. However, trade-offs between GW and other GY components were observed, emphasizing the complexity of GY determination among these cereals. The results also underscore the importance of tailoring N fertilization strategies to specific crops, as triticale and barley showed distinct responses to N levels, with triticale benefiting more from higher N inputs. Future research should focus on exploring the genetic and physiological mechanisms underlying the observed trade-offs between yield components, particularly in two-rowed barley, where low FE and GNS limit GY potential despite high SN and SDWa.

## Figures and Tables

**Table 1:** Basic description of experimental locations in the southern Pannonian Plain.

Location	Novi Sad			Sombor		Sremska Mitrovica	
Coordinate	45°20′ N, 19°51′ E			45°48′ N, 19°07′ E		44°56′ N, 19°49′ E	
Growing season	2019/20	2020/21	2021/22	2019/20	2020/21	2019/20	2020/21
Sowing date	14 October	18 October	27 October	21 October	15 October	9 October	23 October
Harvest date	3 July 2020	29 June 2021	27 June 2022	07 July 2020	28 June 2021	06 July 2020	30 June 2021
Some properties of the soil							
CaCO_3_, %	5.17	5.09	5.37	5.45	6.12	4.30	4.45
P_2_O_5_ (Egner–Riehm), mg/100 g	24.5	23.1	24.9	19.8	17.4	22.5	24.2
K_2_O (Egner–Riehm), mg/100 g	18.4	23.5	20.3	30.1	28.7	23.9	21.8
N (N-min method), kg ha^−1^	85	77	77	75	83	70	90
Humus (Turin)	2.75	2.92	2.89	2.35	2.40	2.48	2.56
pH (KCl)	7.26	7.41	7.35	7.75	7.88	7.35	7.42
Applied P (kg ha^−1^)	70	60	70	80	80	70	60
Applied K (kg ha^−1^)	50	40	50	40	40	40	40

## Data Availability

Data are contained within the article and Supplementary Materials.

## Supplementary Materials

The following supporting information can be downloaded at: https://www.mdpi.com/article/10.3390/plants14071017/s1. Table S1: Grain yield (GY), grain weight (GW), grain number per spike (GNS), grain number per unit area (GN), spike number (SN), spike dry weight at anthesis (SDWa) and fruiting efficiency (FE) of small grain crops across differnt environements (Location × growing season), species, nitrogen treatment and species × nitrogen treatment interaction; Table S2. Characteristics of Tested Cultivars.

## Abbreviations

The following abbreviations are used in this manuscript:

GY	Grain yield
N	Nitrogen
GN	Number of grains per unit area
GW	Grain weight
SN	Spike number per unit area
GNS	Grain number per spike
FE	Fruiting efficiency
SDWa	Spike dry weight per area at anthesis
